# Effect of Si Content on the Thermal Expansion of Ti15Mo(0–2 Si) Biomaterial Alloys during Different Heating Rates

**DOI:** 10.3390/ma16134768

**Published:** 2023-07-01

**Authors:** Hayam A. Aly, Mohamed M. El-Sayed Seleman, Ashraf Bakkar, Ibrahim Albaijan, Mohamed M. Z. Ahmed, Khaled M. Ibrahim

**Affiliations:** 1 Department of Metallurgical and Materials Engineering, Faculty of Petroleum and Mining Engineering, Suez University, Suez 43512, Egypt; hayam.abokhasha79@yahoo.com; 2 Central Metallurgical Research and Development Institute (CMRDI), P.O. Box 87, Helwan 11421, Egypt; khaledabouelela@yahoo.com; 3 Department of Environmental Engineering, College of Engineering at Al-Leith, Um Al-Qura University, Al-Lith 28434, Saudi Arabia; 4 Mechanical Engineering Department, College of Engineering at Al Kharj, Prince Sattam Bin Abdulaziz University, Al Kharj 11942, Saudi Arabia; i.albaijan@psau.edu.sa (I.A.); moh.ahmed@psau.edu.sa (M.M.Z.A.)

**Keywords:** TiMo alloy, Si addition, TiMoSi alloys, biomaterials, casting, dilatometry, thermal expansion, phase transformations, grain refining, microstructure

## Abstract

Thermal expansion measurements were used to characterize phase transformations in metastable β-Ti alloys (Ti15MoxSi) without and with various Si additions (where x = 0, 0.5, 1.0, 1.5, and 2 in wt.%) during linear heating at two heating rates of 5 and 10 °C/min up to 850 °C. For this study, five alloys were developed and examined in terms of their presence phases, microstructures, and starting and final transformation temperatures. According to the results, all of the as-cast samples primarily include an equiaxed β-Ti phase. The influence of phase transformation on the material dimensions was discussed and compared with the variations in Si contents. The transformation was investigated using a dilatometric technique for the developed alloys during continuous heating and cooling. The dilatometric curve of heating revealed two distinct reflection points as the heating temperature increased. The starting transformation temperature (T_s_) to obtain the ω-phase was reported at 359 °C without Si addition; whereas the final transformation temperature (T_f_) of the dissolution of α-phase was obtained at 572 °C at a heating rate of 10 °C/min. At 2 wt.% Si, the first derivative curves reported T_s_ and T_f_ transforming temperatures of 314–565 °C (at a 5 °C/min heating rate) and 270–540 °C (at a 10 °C/min heating rate), respectively. The T_s_ and T_f_ transforming temperatures were significantly decreased with Si additions, which decreased the β-transus temperature. Moreover, the thermal expansion coefficient curves of the investigated alloys without and with 2 wt.% Si were studied. The transformation heating curves have an S-shaped pattern, according to the results.

## 1. Introduction

Titanium (Ti) alloys [1,2,3,4,5], stainless steels [6,7], and cobalt base alloys [8] are the most widely used biomaterials in applications ranging from implants to devices. The range of moduli of elasticity of Ti-based alloys is considerably closer to those of bone [9]. The density of these alloys (4.4–4.5 g/cm^3^) is lower than that recorded by 316L stainless steel (7.9 g/cm^3^) and Co-Cr-based alloys (8.3–9.2 g/cm^3^) [10]. When comparing specific strengths, Ti alloys outperform other metallic biomaterials. Moreover, implants made of Ti alloys may create a thin passive layer of titanium oxide, which represents a spontaneous protection against corrosion or interaction with the surrounding environment [11,12]. The biocompatibility of Ti implants is significantly influenced by their electrochemical and phyco-chemical properties in biological environments [13]. As a result of their low elastic modulus, low density, high strength, good biocompatibility interaction, and high resistance to corrosion, Ti alloys have been used more for higher requirements in biomaterials than traditional alloys. It was found that alloying elements including tantalum (Ta), molybdenum (Mo), zirconium (Zr), and niobium (Nb) are the best for reducing the elastic modulus of cubic Ti while maintaining its strength. Furthermore, these alloying elements are non-toxic, making them suitable for use in implantology [14], whereas aluminum (Al), vanadium (V), chromium (Cr), and nickel Ni are regarded as potentially harmful to human health [15]. Therefore, it is preferable to take biocompatible and safe components into account when designing biomaterials. On the basis of these concerns, researchers have paid more attention to generating and developing new biomaterial Ti based alloys [16,17].

Ti is classified as a polymorphic material. It undergoes phase transformation from a hexagonal close-packed (hcp) structure (α-phase) at ambient temperature to a body-centered cubic (bcc) structure (β-phase) at high temperature of 882 °C [18]. According to this phenomenon, there are three distinct categories for Ti alloys: α Ti-alloys, α plus β Ti-alloys, and β Ti-alloys [3]. These phases can be enhanced by alloying pure Ti or Ti-based alloys with α or β stabilizer alloying elements [3,10]. The majority of research on Ti alloys focuses on the β-alloy type due to the possibility of controlling processing variables to attain specific results in terms of low modulus of elasticity, high corrosion resistance, and enhanced tissue response compared to α plus β alloy types [19,20,21]. These alloys containing this target phase should have enough β-stabilizing elements to preserve the β-phase after quenching [22]. The ability of these Ti alloys to keep the β-phase above β-transus temperature during quenching distinguishes them. Since the retained phase is metastable, the β-Ti alloys’ mechanical performance can be suited by carefully controlling the microstructure during heat treatment. The fine precipitates are dispersed uniformly through the matrix, which is desirable for structural applications. Therefore, understanding phase nucleation and growth kinetics is critical for controlling the resulting microstructure and mechanical properties [23,24,25,26]. The β-transus temperature is essential for heat treatment, especially when heating above or near the β-transus is involved. The transformation process from α-phase to β-phase highly depends on the purity of Ti [27,28]. The change in specimen length caused by temperature determines metal dilatation behavior. When Ti alloys are heated to the β-transus temperature, the crystal structure changes from hcp to bcc, resulting in volume changes and solute atom redistribution. Furthermore, Ti alloys are very similar to steel in transformation from α + β to β phase via heating [29,30]. In fact, phase changes that occur during production and subsequent heat treatment possess a considerable impact on the properties of the metastable Ti alloys.

The dilatometric technique has been widely applied to investigate phase-transformation kinetics. This technique has been successfully utilized by Wan et al. [31] to comprehend phase transformations kinetics in a Ti-1300 alloy at different continuous heating rates and also by Wang et al. [32] to study phase transformation of α plus β phase to β-phase in the TC21 alloy during heating. In addition, Shah et al. [33] applied this method to determine the activation energy of α plus β phase to β-phase transformation in Ti6Al4V alloy. While, a very few studies were performed during continuous heating to clarify the phase transformation form α plus β to β of the important biomaterial TiMo based alloy systems [34,35]. Harcuba et al. [35] related the variation in the electrical resistivity measurements during linear heating of Ti15Mo and Ti6.8Mo1.5Al4.5Fe alloys to the phase transformation.

Based on the previous studies’ findings, no attention has been paid to study phase transformations in Ti-Mo-Si alloys as a new developed biomaterials possessing biocompatibility, improved characteristics, and long-term survivability. As a result, the target of present research is to examine the phase transformation behavior of Ti15MoxSi alloys (where x values vary to be 0, 0.5, 1.0, 1.5, and 2.0 wt.%) at different heating rates of 5 and 10 °C /min. The influence of Si addition on the microstructure features of the Ti15MoxSi alloys was also investigated and discussed.

## 2. Experimental Works

### 2.1. Materials and Preparation

High purity over 99.96 wt.% for Ti, Si, and Mo metals was initially used to develop TiMoxSi alloys. Due to the high melting temperatures of the Ti (1668 °C), Mo (2625 °C), and Si (1414 °C), vacuum arc remelting (VAR) was the method used to attain these biocompatible materials in the Central Metallurgical Research and Development Institute (CMRDI) laboratory to produce the Ti15Mo master alloy and the developed Ti15Mo (0.5, 1, 1.5, and 2) Si alloys. The plasma arc used a high current of 300 A to heat the starting materials selectively. During this method, the Mo melt envelops the Ti to attain the Ti15Mo alloy and the Si pieces to get the (TiMoxSi) alloys. The real benefits of using this process are extremely high melting temperatures, as the electric arc temperature reaches 3300 °C, and the production of near-pure metallurgical atmospheres at a pressure of 700 bar via successive gas purging using high purity (99.9999%) argon gas.

**Figure 1:**
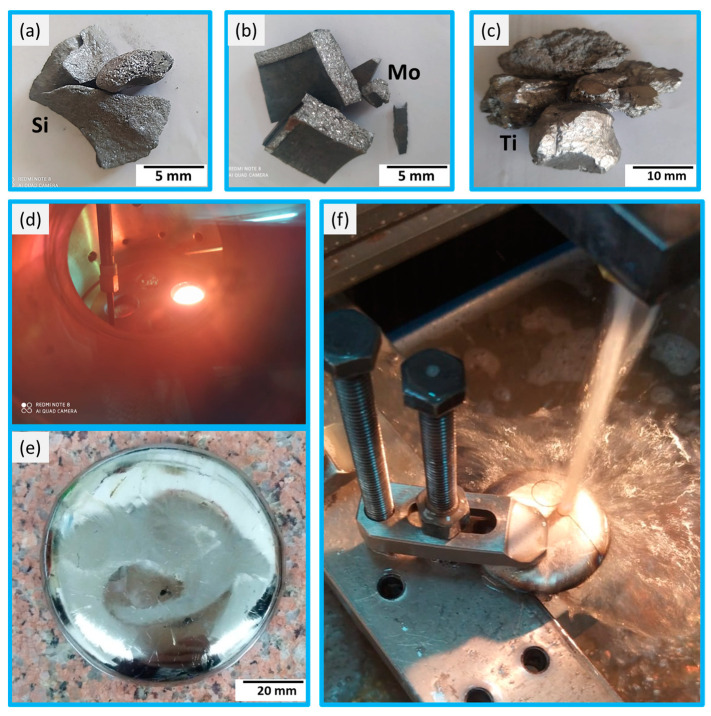
The setup of the processing: (**a**–**c**) are the raw materials of Si, Mo, and Ti, respectively. (**d**) the melting process, (**e**) the final product, and (**f**) the cutting process for different tests.

Before the melting process, the furnace chamber was evacuated by 0.1 bar three times and then flushed with argon to prevent oxidation of the charge. The melting time was adjusted to 80 s, plus 20 s of electromagnetic stirring for each specimen. Six cycles of turning over and remelting each alloy were used to achieve chemical homogeneity, with fast cooling applied to eliminate microscopic segregation of the as-cast materials. This developed casting technique promotes advantages compared to conventional casting techniques [36,37]. The processing setup until the cutting step of the castalloys is demonstrated in Figure 1. The charging calculations for each batch were carried out on the basis of a constant weight (100 g), which was adjusted to the size of the copper mold of the VAR. Table 1 displays the batch design of each Ti-alloy and the efficiency of the melting process.

### 2.2. Chemical Composition, Phase Identification and Microstructure

To examine the chemical composition and constituent phases of the as-cast Ti15Mo and Ti15MoxSi developed alloys, specimens with dimensions of 10 × 10 mm and a 5 mm thickness were cut and polished. Table 2 shows the chemical analyses of as-cast alloys (analyzed by Foundry Master Pro). To obtain precise composition values, five spark determinations were applied in five different areas of the specimens. The X-ray Diffraction (XRD) technique was used to recognize the constituent phases in the cast alloys. The X’PERT PRO. A PANLYTICAL device (Malvern, UK) was used with a Cu-Kα target with secondary monochromatic at 45 kV and 40 mA. The device is computerized in such a way that all necessary information is printed on the computer print sheet. The XRD examination was achieved at a 2θ range from 20 to 100° and a step size of 0.04° to determine the phases present in the developed alloys.

To examine the metallographic structure, the as-cast TiMoxSi samples were prepared as follows: cutting to the required dimensions of 10 × 10 × 5 mm, embedding in an epoxy resin, grinding to a 2500 grit level with emery paper, polishing with an alumina paste to 0.1 µm, and finally chemically etching with a reactive solution (85 mL distilled H_2_O, 5 mL HNO_3_ and 10 mL HF) for 30 sec. After these steps, the specimens were investigated by a scanning electron microscope (SEM) connected with an energy-dispersive X-ray analysis (EDX) system.

To measure the continuous heating and cooling processes, a dilatometer (Linseis L75/230 type, RT-1400 °C, Selb, Germany) connected to an advanced computerized system was used. Specimens of 20 mm in length and 4 mm in diameter were carefully machined from the as-cast alloys using a wire electrical discharge machine (EDM) for the test purpose. The specimens were brought to room temperature in the air after being heated in static air using heating rates of 10 °C/min and 5 °C/min up to 850 °C. Linseis TA software version 2.3.1 was utilized to record the change in length of the specimen during the temperature change. The first derivative was calculated to determine the phase transformation temperature or the thermal expansion coefficient for the as-cast materials using an equation between length and temperature, as discussed later. After the tests, SEM was used again to examine the microstructures.

## 3. Results and Discussion

When Ti-based alloys solidify from the liquid state at high temperatures, cooling rate is considered an essential factor in phase transitions [24,38]. Depending on the cooling rate and alloy composition of the as-cast materials, many solid phases are possible to be formed during the phase transformation process, including β → α, β → α′, β → α″, β → ω, etc. They typically take place at high temperatures and necessitate specific timing. In the current study, all of the produced Ti-alloys were processed using the vacuum arc furnace and subjected to a fast water-cooling system.

Furthermore, as a result of the similarity of the lattice parameters and crystal structures, Mo and Ti may be totally dissolved in each other, resulting in ideal undercooling. This aids in forming the β metastable phase at high temperatures [39]. This means that the β-phase (BCC structure) is attained by adding the transition elements Mo and Si.

**Figure 2:**
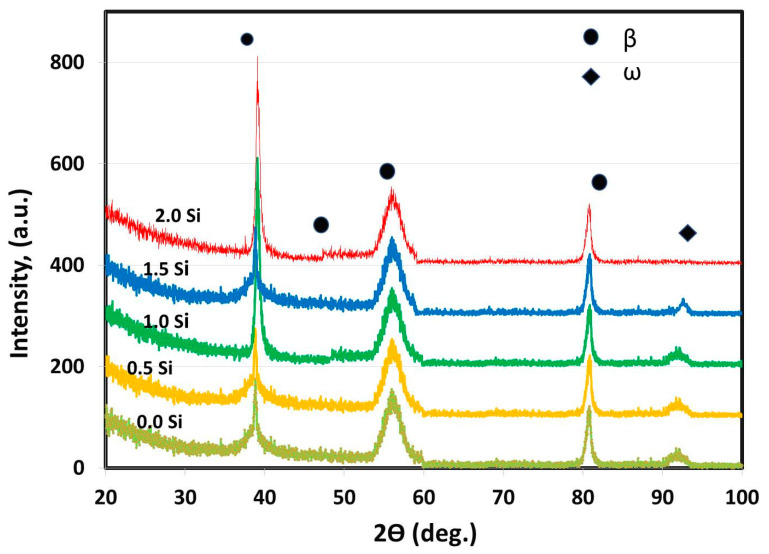
Ti15MoxSi XRD patterns with Si additions of 0, 0.5, 1, 1.5, and 2 wt.%.

For all of the as-cast Ti alloys, the XRD was utilized to recognize the phases existing in each specimen at the lab temperature (25 °C). By analyzing the XRD patterns, it is clear from Figure 2 that only two phases β and ω were detected.

The β-phase constitutes the majority of the samples and is observed in all alloys. This result agreed with the findings of Oliveira et al. [40] as they reported that in the range of 15 to 20 wt.% of Mo addition to the Ti-Mo alloy, only the β-phase exists. In addition, only one small peak of ω-phase is noted with the as-cast master-alloy and that having Si addition of 0.5, 1 and 1.5 wt.%. Both Mo and Si are acting as β-stabilizer elements for Ti [10]. Tavares et al. [41] studied the role of Si addition in Ti35Nb (0, 0.15, 0.35 and 0.05) Si alloy systems subjected to cooling under different conditions. The results showed that as the Si content increased, the density of ω precipitates reduced, which made the β-phase more stable. The ω-phase formation is a significant process for controlling the microstructure in the studied Ti-15Mo system. The ω-phase has hexagonal symmetry and was produced by the described diffusion-less shuffle transformation. The resulting particles are coherent, have the same chemical composition as the β-matrix, and are a few nanometers in diameter [42].

In addition, the XRD results of all specimens are identical, except for the peak intensities and locations of the β-phase and the disappearance of ω-phase at 2 wt.% Si addition. The peak intensity changes with increasing the concentration of the stabilizing elements in the β-phase formed by the solid solution of Si and Mo in the Ti. As a result, the addition of 15 wt.% Mo and 2 wt.% Si may be useful in stopping the formation of ω-phase that slightly decomposes during cooling and contributes to the formation of the only β-phase [42].

**Figure 3:**
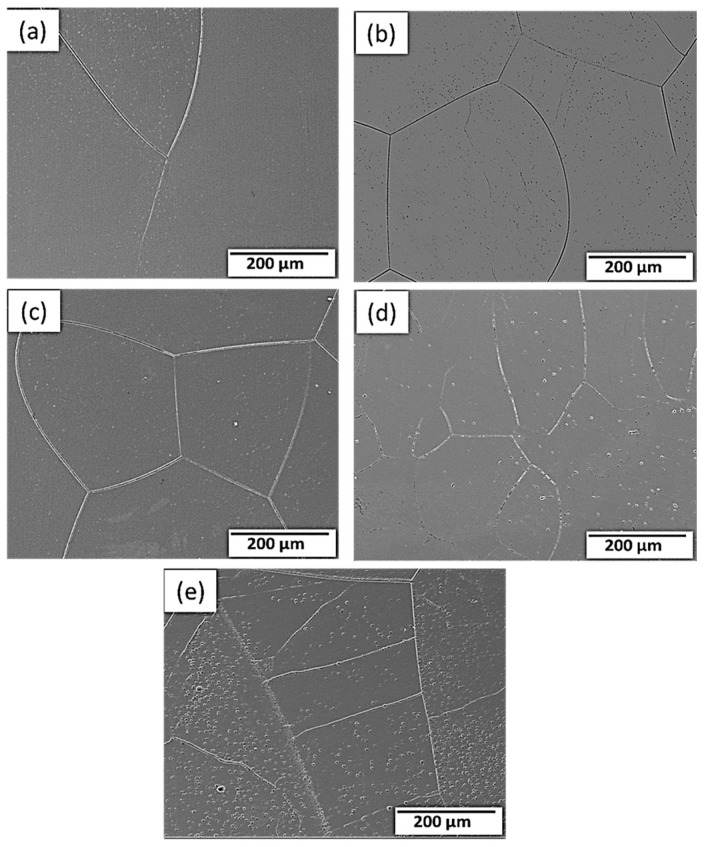
SEM images of the Ti15Mo(0-2)Si alloys with Si additions of (**a**) 0.wt.% Si, (**b**) 0.5 wt.% Si, (**c**) 1 wt.% Si; (**d**) 1.5 wt.%Si, and (**e**) 2 wt.% Si.

**Figure 4:**
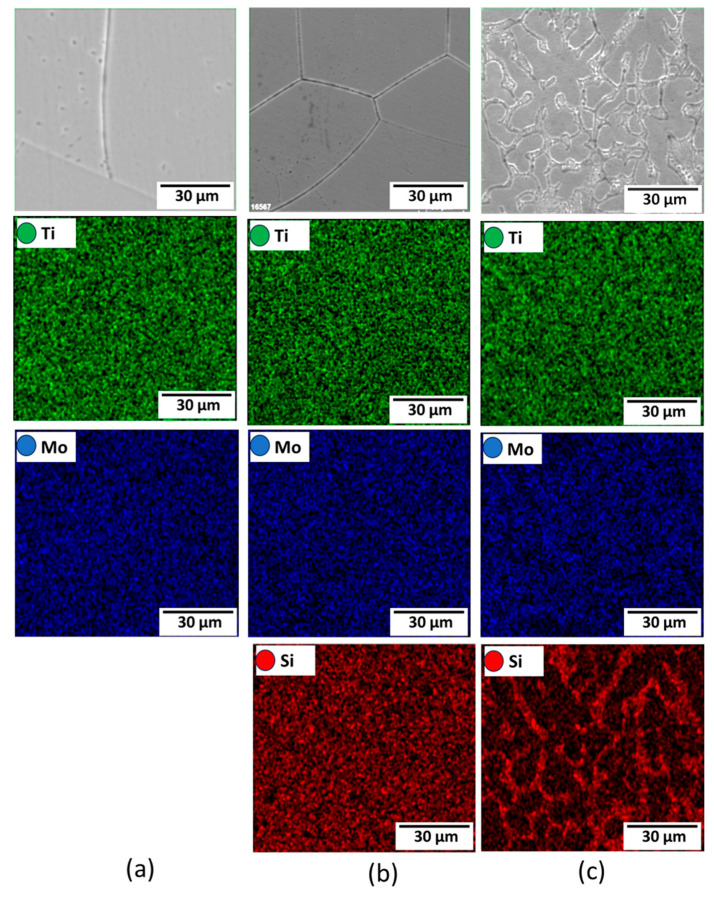
Elemental mapping for (**a**) Ti15Mo, (**b**) Ti15Mo1Si, and (**c**) Ti15Mo2Si alloys.

**Figure 5:**
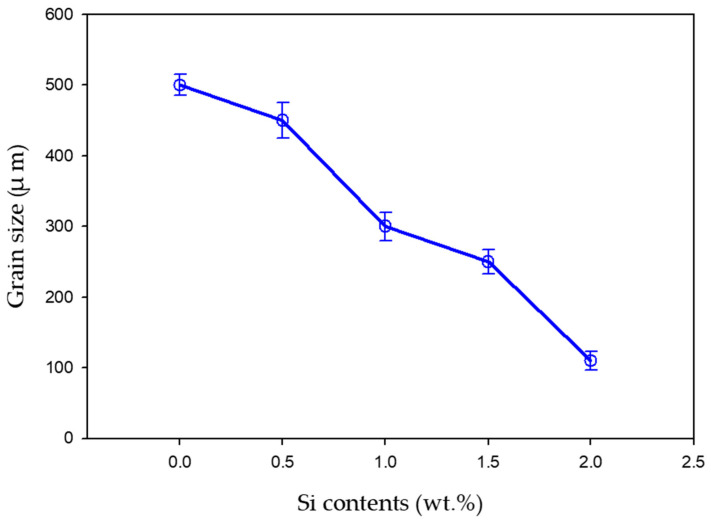
Average grain size of Ti15MoxSi alloys as a function of a Si content (0–2 wt.%).

Figure 3 depicts the microstructural SEM images of the as-quenched Ti15Mo(0–2)Si samples. Figure 4 shows the elemental mapping of the Ti15Mo master alloy, Ti15Mo1Si, and Ti15Mo2Si. As expected, there was only one phase, namely the β-phase. This would imply that the precipitation not observed by SEM in these samples, as well as the tiny white dots, are not inclusions or other phases, but rather the result of pitting due to the chemical etching of the polished surface, as depicted by Figure 3. Without Si addition, the large grains of the β-phase have distinct grain boundaries (Figure 3a). The addition of 0.5 and 1 wt.% Si progressively reduces the particle size of the formed β-phase (Figure 3b,c). When the Si concentration is raised to 2 wt.%, the β-phase’s grain size is the smallest of the five produced cast specimens (Figure 3e). Furthermore, sub-grains are observed in the examined alloys containing 1.5 and 2 wt% Si, as given in Figure 3d,e, respectively. As illustrated by the elemental map in Figure 4c, Si was segregated at grain boundaries of the β-phase. The average grain size of the Ti15MoxSi alloy systems was calculated and plotted in Figure 5 as a function of Si content. It can be remarked that a significant grain refinement was attained with increasing Si content in the Ti15Mo master alloy. The average grain size was 500 ± 15, 450 ± 20, 300 ± 25, 250 ± 15, and 110 ± 13 µm with the Si content 0.0, 0.5, 1.0, 1.5, and 2.0 wt.%, respectively. These findings are in good agreement with previous works [3,20], which reported that grain refining and sub-grains are formed and increased with the increase of Si concentrations.

### 3.1. Dilatometric Analysis

**Figure 6:**
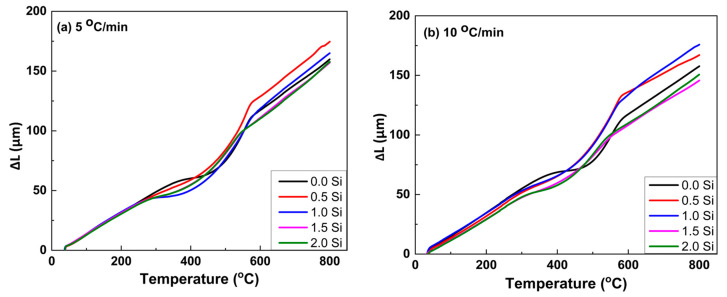
Dilatometric curves, ∆L against temperature, of the Ti15Mo(0-2Si) alloys with applying two different heating rates of (**a**) 5 and (**b**) 10 °C/min.

The thermal expansion of the master alloy (Ti15Mo) and its developed alloys containing Si additions (0, 0.5, 1, 1.5, and 2 wt.%) was measured using a dilatometer at two different linear heating rates of 5 and 10 °C/min up to 850 °C, and the results are plotted in terms of change in length (∆L) versus heating temperatures, as given in Figure 6. For the two heating rates investigated, all curves deviate from linearity. It can be noted that the deviation from linearity begins and ends earlier at the slow heating rate of 5 °C/min than at the faster heating rate of 10 °C/min. At a heating rate of 10 °C/min, the variation in the range of starting transformation temperature (T_s_) and final transformation temperature (T_f_) is shifted to high temperatures. For the master alloy (Ti15Mo), the transformation temperatures T_s_ and T_f_ were 359 and 572 °C, respectively. The transus temperature (T_β_) was 685 °C (when the curves return to the linear trend corresponds to a complete formation of β-phase). But, at the heating rate of 5 °C/min, T_s_, T_f_, and T_β_ were 314, 565, and 673, respectively.

The linear expansion coefficient (α_L_), which represents the derivative of thermal expansion, can provide additional information. The following equation defines the α_L_:

(1) $$\alpha_{L}=\frac{1}{L_{0}}\frac{dL}{dT}.$$

**Figure 7:**
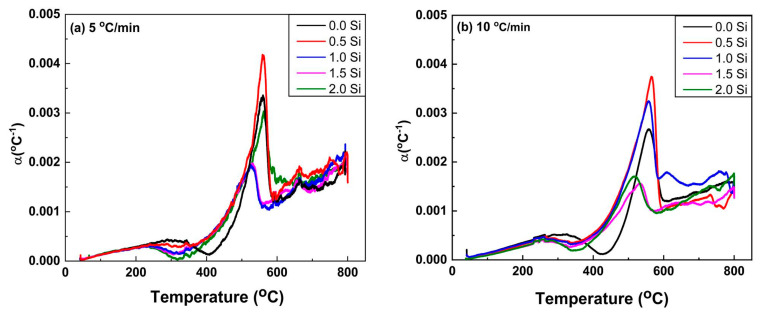
Thermal expansion coefficient of the Ti15Mo(0–2Si) alloys at different heating rates of (**a**) 5 and (**b**) 10 °C/min.

**Figure 8:**
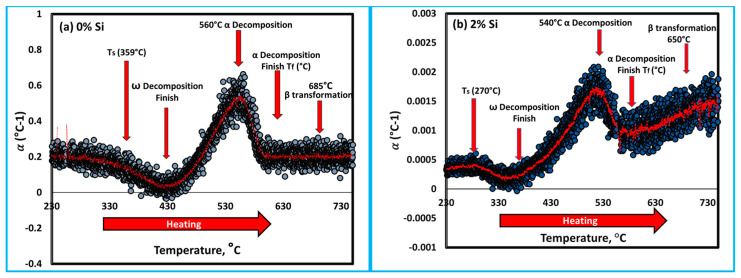
Thermal expansion coefficient of the Ti15Mo master alloy containing different Si content, (**a**) 0 wt.% Si and (**b**) 2 wt.% Si during heating at 10 °C/min.

The α_L_ for both heating rates is shown in Figure 7a,b. It can be indicated that the influence of heating rate on thermal expansion begins slightly earlier at the slow heating rate of 5 °C/min than at the 10 °C/min heating rate. At the heating rate of 10 °C/min, the Ti15Mo2Si alloy obviously shows more influence α_L_ results in the low-temperature range than that thermally heated at 5 °C/min. All curves exhibit the same trends for the thermally tested Ti alloys, as given in Figure 7a,b. Surprisingly, for the temperature ranges from 260 to 410 °C, and 280 to 430 °C, the global minima values of α_L_ were observed, indicating that the material shrinks for the two applied heating rates. This behavior may be explained by a decrease in the ω and β lattice parameters. At high temperatures, α_L_ increases and α particles dissolution at 560 °C (for 0% Si) and 540 °C (for 2% Si) causes an abrupt decrease in α_L._, as shown in Figure 8a,b, respectively. Between 572 and 600 °C (Figure 7), there is a noticeable hump in α_L_ evolution measured at the heating rate of 5 °C/min. This hump is most likely attributed to the subsequent dissolution towards the β-transus and phase formation, as the slow heating rate allows the phase to precipitate for the longest time. At temperatures above 690 °C and for all heating rates, the values of αL are nearly equal, indicating that the material undergoes no further transformations. The last noticed change in α_L_ in the obtained curves, which most likely relates to complete α-phase dissolution (β -transus), takes place at approximately 560 and 540 °C with 0 and 2% Si addition, respectively, see Figure 8.

**Figure 9:**
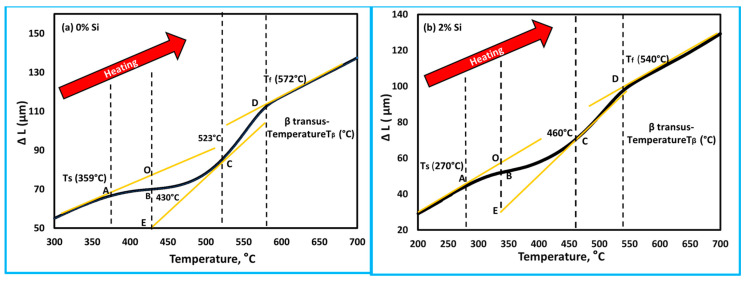
Dilatometry curve in terms of, ∆L against temperature of the as cast Ti-5MoxSi alloy heated at 10 °C/min for (**a**) 0 wt.% Si and (**b**) 2 wt.% Si. The black lines are the ∆L against temperature curve and the yellow lines are tangents indicating phase transformation.

To understand the role of Si addition on the behavior of the thermal properties of the developed cast alloys; the Ti15Mo and Ti15Mo2Si alloys were separately compared depending on the results obtained in the dilatometry curves (Figure 6b) for the specimens heated at a heating rate of 10 °C/min, as seen in Figure 9. At the beginning of linear heating, Ti15Mo and Ti15Mo2Si alloys dilate linearly as a result of thermal expansion of the β-phase crystal lattice. With heating to point A, 359 °C (for Ti15Mo) and 270 °C (for Ti15Mo2Si), the two alloys begin to shrink as the slope of the curves slightly decreases due to the detrimental dilatation influence of the ω-phase precipitation [42]. The yellow line AO represents the tangent line to the starting ω-phase transformation curve. The dilatometry curve then continuously decreases as the β → ω phase transformation proceeds, reaching its minimum at 430 °C for 0% Si and 340 °C for 2% Si as given by point B in Figure 9a,b, respectively. With further temperature raising, the ω-phase transformation ended, and the α-phase began to nucleate in the ω-phase core [43]. The finishing ω phase transformation curve is represented by the yellow tangent EC. Due to this phase transformation, the length of the sample dilates rapidly. When the alloy is heated to elevated temperatures, an intergranular and conventional intragranular phase begin to precipitate at 523 °C for 0% Si alloy and 460 °C for 2% Si alloy, as given by point C in Figure 9a,b, respectively. This followed by a slow increase in the curve to reach point D (end of α-phase transformation and start β-phase formation with further heating).

**Figure 10:**
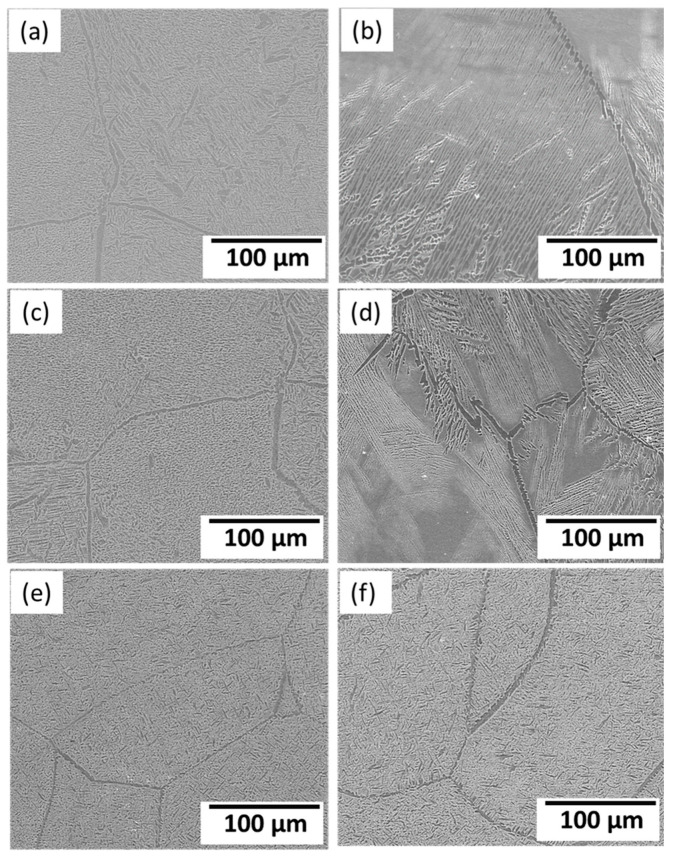
SEM images of the Ti15MoxSi casted alloys after dilataion at different heating rates: (**a**) x = 0 wt.% at 5 °C/min; (**b**) x = 0 wt.% at 10 °C /min; (**c**) x = 1 wt.% at 5 °C /min; (**d**) x = 1 wt.% at 10 °C /min, (**e**) x = 2 wt.% at 5 °C /min, and (**f**) x = 2 wt.% at 10 °C /min.

**Figure 11:**
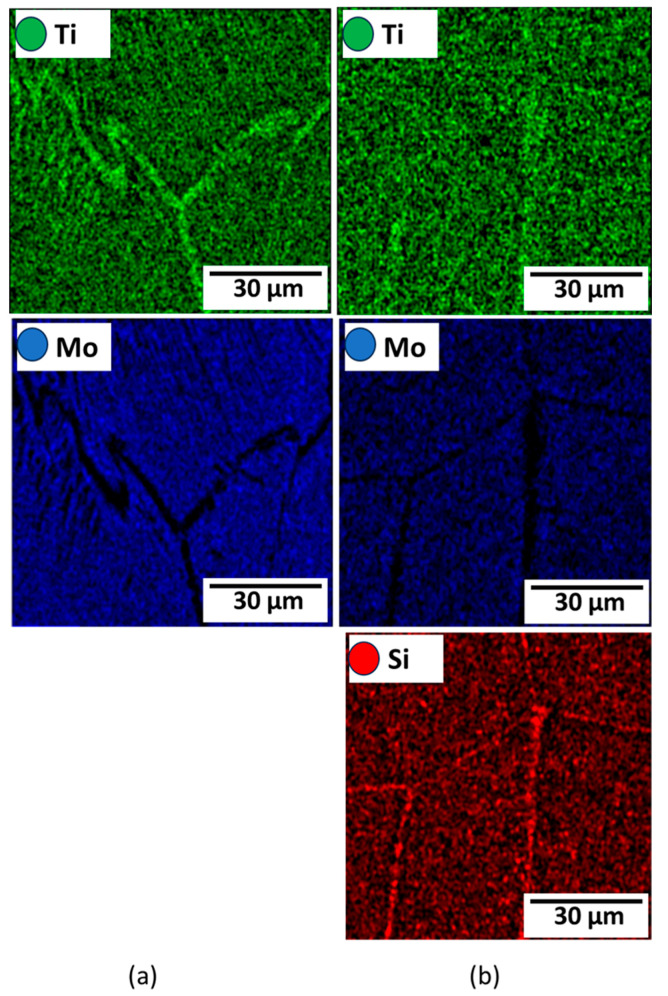
Elemental mapping of (**a**) Ti15Mo and (**b**) Ti15Mo2Si alloys.

The dilatometry curves of the specimens subjected to a heating rate of 5 °C/min and xSi can also be divided into four regions (Figure 6a). Table 3 illustrates the transformation temperatures Ts, T_f_ and T_β_ of the different investigated Ti-base alloys at 5 and 10 °C/min heating rates. It can be noted that increasing the heating rate causes these transformation temperatures of the as-cast Ti alloys to shift to higher temperature values. In addition, the increases in Si content in the Ti15Mo mater alloy enhance the transformation temperatures of Ts, T_f_ and T_β_ to occur at lower temperatures. Hence, it can be said that the addition of 15 wt.% Mo and 2 wt.% Si elements to Ti alloy might be useful in avoiding the formation of ω-phase, and α-phase in the produced alloy. Figure 10 and Figure 11 give the SEM micrographs of the Ti15MoxSi casted alloys at different heating rates and the mapping of the Ti15M and Ti15Mo2S alloy samples, respectively.

The variations in the microstructure feature in terms α-phase content and its morphology after dilatation are governed by the applied cooling rate and alloy chemical composition [31,34], as shown in Figure 10. The greater the quantity of α-phase to form, the slower the cooling rate applied. Furthermore, α-phase appears as needle-like, as shown in Figure 10b,d,f. The SEM- mapping images represent the homogeneity of alloying elements Mo and Si in Ti alloy with the presence of needles, as shown in Figure 11.

In brief, at room temperature, the Ti15MoxSi quenched materials (where x: 0, 0.5, 1 and 1.5 wt.%) contain mainly a β-phase matrix, as represented with narrow ω peaks at this temperature (see Figure 2) as a result of the precipitation of tinny particles of ω phase [22]. The ω-phase generated during fast cooling is known as athermal ω. It is coherent with the β-phase with sharing the same chemical composition. This phase disappears completely for the quenched Ti15Mo2Si alloy, indicating the action of high content Si in hindering of ω-phase formation. Besides, the addition of Si reduces the grain sizes in the microstructure of the Ti15Mo alloys. The grain size of the Ti15Mo2Si alloy was reduced to be 110 ± 13 µm compared to 500 ± 15 µm for the master alloy Ti15Mo. This reduction in grain size improves the mechanical properties. It can be noted that dilatometry curves of as-cast Ti alloys shift to higher temperature ranges as heating rates increase from 5 to 10 °C/min during the continuous heating process. The Si addition decreases the transformation temperatures of T_s_, T_f_, and T_B_ for the Ti15Mo(0–2 wt.%)Si at these two applied heating rates (Table 3). At a 5 °C/mm heating rate, the T_s_,T_f_, and T_B_ reached at 314, 565 and 673 °C for the Ti15Mo master alloy without Si addition, while these values for the Ti15Mo2Si alloy are 260, 530, and 640 °C, respectively. In the case of using a 10 °C/mm heating rate, the transformation temperatures of T_s_, T_f_, and T_B_ become 270, 540 and 650 °C for the alloy containing 2 wt.% Si compared to those recorded by the alloy without Si addition at 359, 572 and 685 °C, respectively.

Since Si has superior biocompatibility and is considered a β-stabilizer, it was utilized as an alloying element in all of the as-cast Ti-15Mo specimens. The addition of Si can improve the transformed temperature to obtain the β-phase significantly. The intensity of the β transus-temperature Tβ (°C) is attained by increasing content of the stabilizing elements in the β-phase formed by the solid solution of Si in Ti. As a result, the addition of Mo and Si elements may effectively hinder the ω-phase formation that slightly decomposes during the cooling process and contributes to the presence of the β-phase at low temperatures.

### 3.2. Phase-Transformation Kinetics

The Ti base alloys, as polycrystalline materials, exhibit isotropic dilatation properties during the solid-state phase transformation. Therefore, the relation between the change in volume (∆V/V_o_) and the relative length (∆L/L_o_) can be given in the following equation [31,32]:

(2) $$\frac{\Delta V}{V_{o}}=\frac{3\Delta L}{L_{o}}.$$

**Figure 12:**
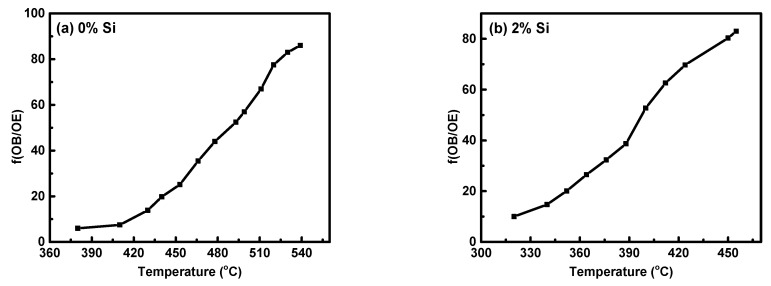
Volume fraction of ω-phase transformed versus temperatures for Ti15MoxSi alloys: (**a**) x = 0% Si and (**b**) x = 2% Si.

L_o_ denotes the original length of specimen, V_o_ denotes the original volume, and ΔL and ∆V denote the variations in length and volume, respectively. As previously stated, the ω-phase precipitation is responsible for the non-linearity of the dilatometry curve in the A-B domain (Figure 9). When considering the examined Ti15MoxSi specimen dilatometric heating curve, the deviation degree of the dilatometric curve ought to be proportionate to the β → ω volume fraction that has been transformed. As a result, the lever rule can be used to investigate the relationship between the heating temperature and the β → β + α transformed volume fraction (ƒ_ω_) (Figure 12a,b) with and without Si addition can be expressed by.

(3) $$f_{\omega }=\frac{L_{OB}}{L_{OE}}.$$

where L_OB_ and L_OE_ are the lengths of the OB and OE lines, as shown in Figure 9. Figure 12 depicts the transformation curve of β → β + α, which displayed a regular pattern of S-shaped. This indicates that the as-cast alloys’ β → β + ω + α phase transformation has a nucleation growth-controlled process [44,45]. These results are consistent with that reported by Wan et al. [31] and Hua et al. [46]. The addition of Si can significantly enhance the ω-phase restraining and its finished transformed temperature, which occur at 523 °C for 0% Si and 460 °C for 2% Si at a heating rate of 10 °C/min.

## 4. Conclusions

The impact of Si addition on the thermal expansion of Ti15Mo(0–2)Si quenched alloys at various heating rates of 5 and 10 °C/min was investigated. Dilatometry proved to be an effective tool for detecting phase transformations in Ti alloys. The following are the main findings of the current study:

- The dilatometric heating curve at a 10 °C/min heating rate revealed that the starting T_s_ and T_f_ of Ti-15Mo master alloy were 359 °C and 572 °C, respectively, whereas the T_s_ and T_f_ transforming temperatures were significantly decreased with 2 wt.% Si additions, reaching 270 °C and 540 °C, respectively.
- Increasing the Si content of the Ti15Mo master alloy to produce Ti15Mo(0–2 wt.%Si) alloys resulted in a significant grain refinement. The average grain size was 500 ± 15, 450 ± 20, 300 ± 25, 250 ± 15 and 110 ± 13 µm with the Si addition of 0.0, 0.5, 1.0, 1.5, and 2.0 wt.%, respectively.
- The β-transus temperature of Ti15Mo(0–2 wt.%Si) alloy systems slightly decreases with increasing the Si content for the two studied heating rates. In the case of using a heating rate of 10 °C/min, this transformation temperature happens at about 650 °C with the Si content 2 wt.% which is lower than the transus-temperature of the common Ti β alloy at ≈882 °C.
- From dilatometry curves, it can be concluded from the phase transformations in the continuous heating process that phase precipitation occurs first in domains A-B, and is followed by phase dissolving in domains B-C.
- Using the lever rule, we estimated the ω-phase transformation curve in Ti15MoxSi alloys under continuous heating. Our results show an S-shaped pattern, with the “S” shifting to a lower temperature range as the Si addition increases.

## Figures and Tables

**Table 1:** Batch design and melting process efficiency of Ti15MoxSi alloys of different Si content (in wt.%).

Alloy Composition	Element, (g)			Batch wt., (g)	Ingot wt., (g)	Efficiency, (%)
	Ti	Mo	Si			
Ti15Mo	85.00	15.01	-	100.01	99.90	99.89
Ti15Mo0.5Si	84.50	15.00	0.50	100.00	99.98	99.98
Ti15Mo1.0Si	83.90	15.13	1.01	100.04	100.01	99.97
Ti15Mo1.5Si	83.50	15.04	1.53	100.07	100.00	99.93
Ti15Mo2.0Si	82.90	15.11	2.12	100.13	99.96	99.83

**Table 2:** Chemical composition of the Ti15Mo(0–2)Si casted alloys (in wt.%).

Batch wt., (g)	Ti	Mo	Si	Fe	Mn
Ti15Mo	84.947	15.030	-	0.003	0.020
Ti15Mo0.5Si	84.475	15.000	0.520	0.003	0.002
Ti15Mo1.0Si	83.866	15.100	1.010	0.002	0.022
Ti15Mo1.5Si	83.367	15.120	1.510	0.004	0.002
Ti15Mo2.0Si	82.867	15.090	2.030	0.011	0.002

**Table 3:** The transformation temperatures Ts, T_f_ and Tβ of different investigated Ti-base alloys at 5 and 10 °C/min heating rates.

Alloys	5 °C/min			10 °C/min		
	T_s_ (°C)	T_f_ (°C)	β Transus–Temperature Tβ (°C)	T_s_ (°C)	T_f_ (°C)	β Transus–Temperature Tβ (°C)
Ti15Mo	314	565	673	359	572	685
Ti15Mo0.5Si	295	559	671	330	580	680
Ti15Mo1.0Si	283	551	664	290	560	675
Ti15Mo1.5Si	271	542	660	275	550	665
Ti15Mo2.0Si	260	530	640	270	540	650

## Data Availability

Data will be available upon request through the corresponding author.
